# Evaluation of effectiveness of palliative radiotherapy for bone metastases: a prospective cohort study

**DOI:** 10.1007/s13566-018-0363-6

**Published:** 2018-11-10

**Authors:** Joanne M. van der Velden, Yvette M. van der Linden, Anne L. Versteeg, Jorrit-Jan Verlaan, A. Sophie Gerlich, Bart J. Pielkenrood, Nicolien Kasperts, Helena M. Verkooijen

**Affiliations:** 1 Department of Radiation Oncology, University Medical Center Utrecht, Heidelberglaan 100, 3584 CX Utrecht, The Netherlands; 2 Department of Radiation Oncology, Leiden University Medical Center, Albinusdreef 2, 2333 ZA Leiden, The Netherlands; 3 Department of Orthopedic Surgery, University Medical Center Utrecht, Heidelberglaan 100, 3584 CX Utrecht, The Netherlands; 4 Department of Clinical Epidemiology, Julius Center for Health Sciences and Primary Care, Universiteitsweg 100, 3584 CG Utrecht, The Netherlands

**Keywords:** Bone metastases, Palliative radiotherapy, Effectiveness, Prospective cohort study

## Abstract

**Objective:**

Radiotherapy is the standard local treatment for patients with painful bone metastases, but effectiveness has primarily been evaluated in trial populations. The aim of this study was to study pain response to palliative radiotherapy in a prospective cohort of unselected patients with bone metastases.

**Methods:**

Patients with painful bone metastases referred to the UMC Utrecht for radiotherapy and enrolled in the PRESENT cohort were included in this study. For all patients, pain response to radiotherapy was assessed, and responders were defined as patients with a complete or partial pain response. Patients with stable pain scores, pain increase, or undetermined response were regarded non-responders. Pain scores obtained at baseline and after 2, 4, 6, 8, and 12 weeks following radiotherapy were obtained. Pain response rates of the total treated population, as well as response rates of the assessable patients, were calculated. To measure the percentage of the remaining time spent with pain relief, the net pain relief (NPR) was calculated by dividing the period of pain relief by the period of survival.

**Results:**

Of the 432 patients enrolled in this study, 262 patients (61%) experienced a complete or partial response. In the 390 assessable patients, this percentage was 67%. Median time to response was 4 weeks (range 1–15 weeks), and the NPR was 64%.

**Conclusion:**

Compared to randomized trial populations, palliative radiotherapy in our unselected patients with bone metastases showed similar pain response rates (61%), with a reasonable duration of this effect.

## Introduction

Many patients with cancer develop bone metastases, with pain as a common and severe consequence. Due to improved treatment options for primary tumors and subsequent longer survival, the number of patients with bone metastases is likely to increase. They represent a heterogeneous group with differences in tumor histology, extent of the disease, and expected survival. As the diagnosis of bone metastases often represents incurable disease, the main treatment goal is palliation of symptoms [1, 2]. Palliative radiotherapy constitutes the standard of care for patients with painful bone metastases, sometimes combined with other treatments such as a change in pain medication, radiopharmaceuticals, or surgical stabilization. The best evidence regarding the effectiveness of treatments is generally provided by randomized controlled trials. Systematic reviews of palliative radiotherapy trials for bone metastases showed a similar overall pain response rate in patients receiving single versus multiple fractions with pooled pain response rates of approximately 60% [3, 4]. Clinical trials usually use strict inclusion criteria, resulting in the inclusion of selected patients, who are often not representative of the entire patient population. Restricted patient access to trials and physician or patient resistance to randomization might lead to further selected recruitment [5]. What is more noteworthy, trials in the metastatic setting or trials investigating radiation treatments are more likely to be classified as trials with slow recruitment, thereby increasing the risk of selected enrollment [6]. This limits the generalizability of the results of these trials as patients enrolled in clinical trials—also in trials investigating palliative research questions—are usually a (relatively) healthier subgroup of patients referred for treatment [7, 8]. Therefore, our aim was to study whether pain response rates from randomized trials are similar to those observed in patients with bone metastases treated in daily practice.

## Methods

Patients were retrieved from the prospective PRospective Evaluation of interventional StudiEs on boNe meTastases (PRESENT) cohort, which includes all patients with (un)complicated bone metastases referred to the departments of radiation oncology or orthopedic surgery at our center since June 2013 [9]. The PRESENT cohort follows the cohort multiple randomized controlled trial design and serves as an infrastructure for efficient and pragmatic (randomized) treatment evaluation [10]. In this context, patients give separate informed consent to be offered experimental interventions at random [11]. At enrollment, written informed consent for the collection of baseline demographics, treatment characteristics, and clinical follow-up data is obtained. Patients are also asked to provide patient-reported outcomes by filling out the Brief Pain Inventory (BPI, [12]), the EORTC QLQ-C15-PAL [13], the EORTC QLQ-BM22 [14], and the EQ-5D [15] at baseline and 2, 4, 6, and 8 weeks, and 3 and 6 months after the initial radiotherapy and every 6 months thereafter. The PRESENT study was approved by the Institutional Review and Ethics Board of the UMC Utrecht, The Netherlands.

This study was performed after enrollment of the first 500 PRESENT patients. For the present analysis, we excluded patients who did not undergo radiotherapy (*n* = 9), patients not having metastases from solid tumors (*n* = 18), patients who underwent stereotactic radiotherapy (*n* = 25), and patients with asymptomatic lesions (*n* = 27). At our department, patients in good clinical condition with a limited number of bone metastases from favorable tumors are usually treated with long course radiotherapy—with 30 Gy in 10 fractions being the most common long course schedule—but the default radiotherapy schedule is single fraction radiotherapy of 8 Gy.

The primary endpoint was pain response between 2 and 12 weeks after radiation treatment. Patients reported their pain score on a scale from 0 (no pain) to 10 (worst imaginable pain), and the BPI item *worst pain in the last 3 days* was used. In addition, analgesic use was recorded, and all opioid analgesics were expressed as the oral equivalent daily morphine use. According to the international consensus criteria [16], complete response was defined as a pain score of 0 without an increase in analgesic use. Partial response was defined as pain reduction of at least two points without an increase in analgesic use, or at least a 25% reduction in opioid use without an increase in pain score. Patients were classified as responders if a complete or partial response was achieved on at least one of the follow-up time points. All other patients were classified as non-responders. In case a patient did not return the questionnaires, a research nurse contacted the patient by phone or the patient’s medical records were consulted for notes of the telephone contact with the doctor during follow-up. The date of return of the questionnaire or date of telephone contact was recorded. For performance status, either WHO or KPS score was recorded. KPS scores were converted into WHO scores for uniform reporting [17]. Survival data were obtained through the population registry until June 5, 2017. For patients with multiple treatment fields, only lesions treated at baseline were taken into account. Patients with a response for one lesion, but not for another were regarded non-responders.

### Statistical analysis

Descriptive statistics were provided as percentages for proportions, mean and 95% confidence intervals (95% CI), or median and ranges for continuous values. Several proportions of response were calculated: the response rate in the total treated population, the response rate in the population surviving at least 2 weeks after treatment, and the response rate in the assessable population (i.e., all patients who reported pain scores). In addition, two sensitivity analyses were performed: first, a worst-case scenario analysis, assuming all patients without information on pain response to be a non-responder; and a second analysis, in which patients without information on pain response were assumed responders (“best case scenario” analysis). To explore the influence of the time frame on the pain response rate, pain response was calculated between 4 and 8 weeks following radiotherapy. Pain response rates were assessed for three subgroups: in patients with breast or prostate cancer, in patients with spinal metastases, and in patients in good physical condition. Time to response was measured from date of treatment to date of first response. Survival was measured from date of radiation treatment to date of death or last contact. Response duration was calculated in days in patients who experienced pain relief from the first evaluable date of response to the date of relapse (defined as an increase in pain or analgesic use score as compared to baseline), or in absence of relapse to the date of last assessment or death. Retreatment was considered relapse. To measure the percentage of remaining time spent with pain relief, the net pain relief (NPR) was calculated by dividing the period of pain relief by the period of survival in days and multiplying the result by 100 [16, 18]. Retreatment rate was defined as the proportion of patients receiving re-irradiation or surgery at least 4 weeks after the initial treatment. All statistical analyses were performed using IBM SPSS statistics version 23.0 (IBM Corp., Armonk, NY, USA). We reported our results according to the Strengthening the Reporting of Observational Studies in Epidemiology (STROBE) guidelines [19].

## Results

**Fig. 1:**
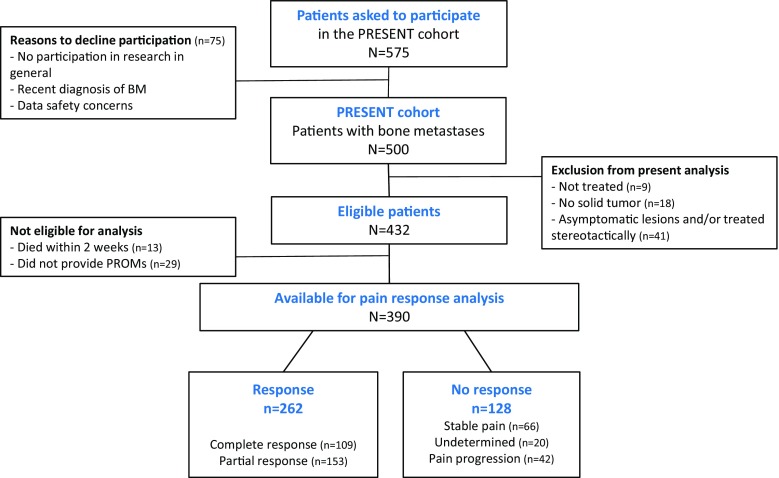
Study flow chart

Of all patients who were invited for the PRESENT study, 87% agreed to participate (Fig. 1). Five hundred patients were enrolled between June 2013 and August 2015. Of these, 432 patients were eligible for the present analysis. At baseline, 345 patients (80%) were treated for 1 painful lesion, 73 patients (17%) were treated for 2 lesions, 36 patients (2.5%) for 3 lesions, and 2 patients (0.5%) were treated for 4 lesions. The spine was the most affected site (64% of all treatment sites) (Table 1). Most common primary cancer sites were prostate (29%), breast (23%), and lung (23%). During radiotherapy, 147 (34%) of the patients used corticosteroids for various reasons including neurological complaints, antitumor treatment, and prevention of pain flare.

**Table 1:** Characteristics of the first 500 patients in the PRESENT cohort

	Entire cohort, *N* = 432	Non-assessable patients^a^, *N* = 42
Gender		
Male	255 (59%)	27 (64%)
Female	177 (41%)	15 (36%)
Age		
Median (range)	67 (28–90)	64 (49–79)
Primary cancer site		
Prostate	127 (29%)	6 (14%)
Breast	97 (23%)	5 (12%)
Lung	97 (23%)	19 (45%)
Other	111 (25%)	12 (29%)
Localization^b^		
Spine	302 (63%)	40 (72%)
Pelvis	98 (20%)	11 (20%)
Long bones	33 (7%)	1 (2%)
Ribs	12 (3%)	1 (2%)
Other	35 (7%)	2 (4%)
Radiation treatment		
8 Gy; 1 × 8 Gy	290 (67%)	50 (86%)
30 Gy; 10 × 3 Gy	72 (17%)	3 (5%)
Other	70 (16%)	5 (9%)
Visceral and/or brain metastases		
Visceral	170 (39%)	22 (52%)
Brain	6 (1%)	0
Both visceral and brain	6 (1%)	2 (5%)
WHO performance status^c^		
WHO 0–1	202 (53%)	9 (26%)
WHO 2	146 (39%)	19 (54%)
WHO 3–4	32 (8%)	7 (20%)
Pain score		
Mean ± SD	6.4 ± 2.2	6.5 ± 2.6
Median (range)	7 (0–10)	6.5 (0–10)
Pain medication^d^		
No use	36 (8%)	0
Phase 1 or 2	130 (30%)	7 (17%)
Phase 3 or 4	264 (62%)	34 (81%)
Use of corticosteroids^e^		
No	269 (65%)	21 (51%)
Yes, during radiotherapy	18 (4%)	3 (7%)
Yes, for neurological complaints	29 (7%)	2 (5%)
Yes, for other reasons	53 (13%)	11 (27%)
Yes, for unknown reasons	47 (11%)	4 (10%)

^a^Patients not returning questionnaires during follow-up

^b^In total, 73 patients had two or more lesions at baseline

^c^The WHO is a conditional score ranging from 0 (normal situation, no complaints) to 4 (completely disabled). Of 52 patients (12%), WHO status was missing

^d^Pain medication phase 1: non-opioids like paracetamol, NSAIDs; phase 2: mild opioids like tramadol; phase 3: strong opioids like morphine; phase 4: non-oral administration of opioids. Data on pain medication was missing in two patients (0.004%)

^e^For corticosteroid use, data of 16 patients (4%) were missing

During a median follow-up of 3 months, 89 patients (21%) developed new painful lesions outside the initial radiation target volume, which were subsequently irradiated. Median time to irradiation of these new lesions was 17 weeks (range 2–80 weeks). Of these 89 patients, 36 patients (40%) developed new painful lesions within 12 weeks after irradiation of the baseline lesion. Twenty patients (55%) experienced a response before they were treated for a new lesion, 5 patients (14%) did not respond before treatment of the new lesion, and 9 patients (25%) had progressive disease.

The 42 patients who did not return the pain questionnaires were different from the rest of the cohort participants; they had more often lung cancer as a primary tumor, were more often treated with single fraction radiation therapy, and their physical condition was worse compared with patients who returned the questionnaires (Table 1). Thirteen patients (3%) died within 2 weeks after radiation treatment, and 117 patients (27%) died within 3 months. For the entire cohort, median survival was 8 months (range 0–46 months).

### Pain response

Response rates of 390 patients (90%) were available (Table 2). Response rates were based on returned questionnaires (71%) or follow-up phone call (29%). In the total treated population, 262 patients experienced a response (61%, 95% CI 56–65%). Patients treated with long course radiotherapy experienced pain relief more often compared to patients treated with 8 Gy in a single fraction (72 vs. 55% respectively, *p* = 0.001). Retreatment rate for the entire cohort was 24%, with no significant difference between the patients receiving 8 Gy in a single fraction (26%) and patients receiving long course radiotherapy (20%, *p* = 0.223). Median time to re-irradiation, however, was significantly different between the two groups (17 weeks vs. 81 weeks respectively, *p* = 0.000). The response rate in the population surviving at least 2 weeks after treatment was 63% (95% CI 58–67%). Of all assessable patients, the response rate was 67% (95% CI 62–72%). The two scenario analyses showed different outcomes. In the worst-case scenario analysis, the proportion of response was 61% (i.e., the proportion of responders in the total study population). In the scenario in which patients with an unknown response were regarded responders, the response rate was 70% (95% CI 66–75%). The period in which pain response rate was recorded was of the influence of the response rate. The response rates between week 4 and week 8 was 51% (95% CI 46–56%) for the total treated population (worst case scenario), and 62% (95% CI 57–67%) for the assessable patients, which is lower than the response rates measured in week 2 to week 12. Median time to response was 4 weeks (range 1–15 weeks). When patients experienced pain relief, the median duration of pain response was 21 weeks (range 0–190 weeks). The NPR in this population was 64% (95% CI 60–66%). The median survival between patients with and patients without a pain response differed significantly (13 vs. 5 months respectively, *p* < 0.001).

**Table 2:** Best response outcomes in 2–12 weeks after radiotherapy according to the consensus criteria [16]

Response type	Total treated population (*n* = 432)	All patients surviving at least 2 weeks patients (*n* = 419)	Assessable patients (*n* = 390)	Worst case scenario (*n* = 432)	Best case scenario (*n* = 432)
Responders	262 (61%)	262 (63%)	262 (67%)	262 (61%)	304 (70%)
Complete response	109 (25%)	109 (26%)	109 (30%)	109 (25%)	NA
Partial response	153 (36%)	153 (36%)	153 (38%)	153 (36%)	NA
Non-responders	128 (29%)	128 (30%)	128 (33%)	170 (39%)	128 (30%)
Stable pain	66 (16%)	66 (16%)	66 (17%)	NA	NA
Undetermined^a^	20 (5%)	20 (5%)	20 (5%)	NA	NA
Pain progression	42 (10%)	42 (10%)	42 (10%)	NA	NA
Unknown	42 (10%)	29 (7%)	NA	NA	NA

^a^Response not captured by response, stable pain, or pain progression, for example, a patient with decreasing pain scores with simultaneously increasing opioid use

### Subgroup analyses

**Fig. 2:**
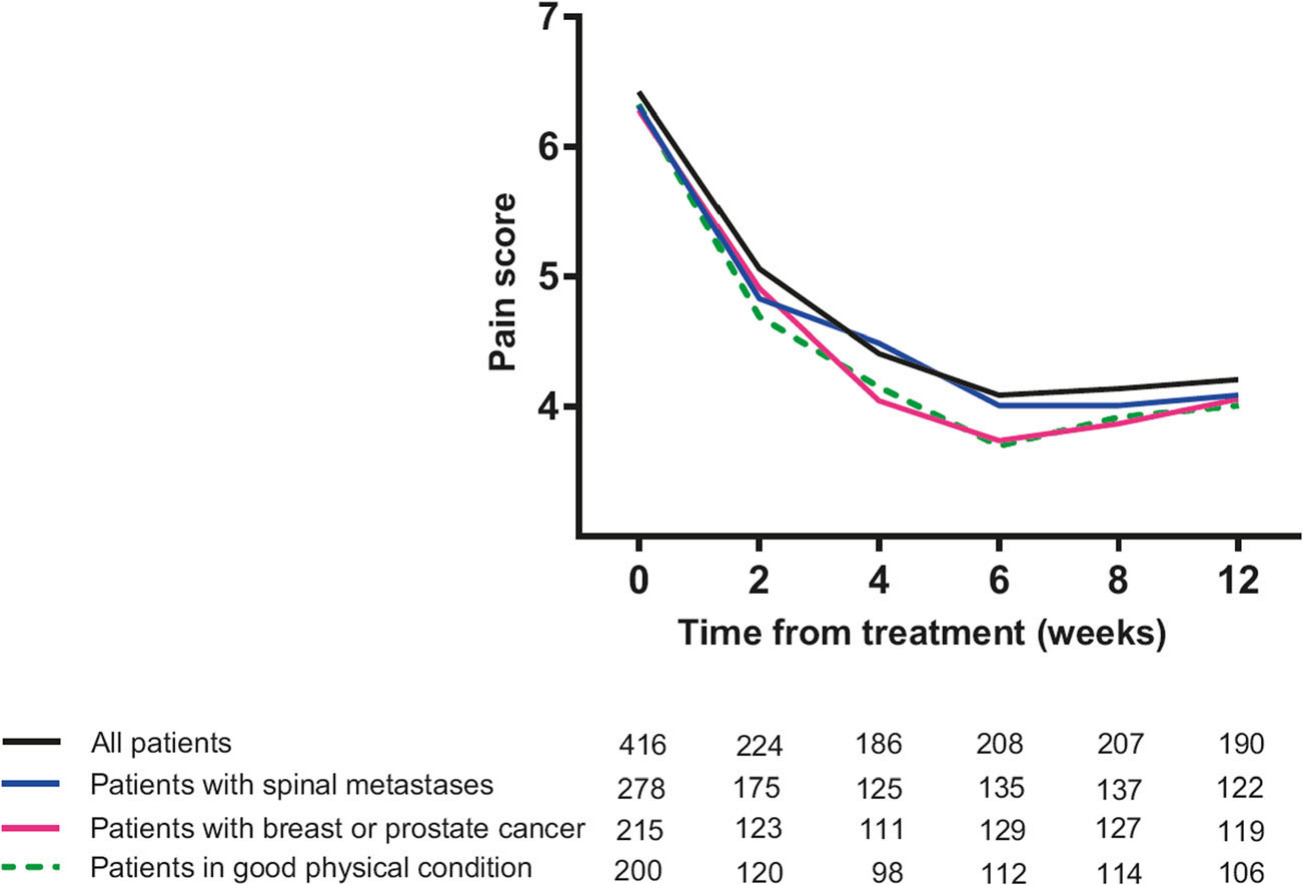
Pain scores during the first 12 weeks after treatment for all patients, patients with spinal metastases, patients with breast or prostate cancer, and patients in good clinical condition (i.e., *WHO score 0*–*1*). Pain was scored on a 11-point pain scale ranging from 0 (no pain) to 10 (worst imaginable pain). The numbers below the graph indicate the number of patients that provided pain scores at specific time points

Of all patients with spinal metastases, 60% (95% CI 54%–65%) experienced a pain response (Table 3). Patients with breast or prostate cancer (*n* = 224) and patients in good physical condition (*n* = 202) had higher response rates of 70% (95% CI 64%–76%) and 69% (95% CI 63%–76%), respectively (Table 3). For the entire cohort and all subgroups, pain scores were lowest at 6 weeks after radiotherapy, after which mean pain scores increased again but were still lower compared to baseline (Fig. 2).

**Table 3:** Best response outcomes in 2–12 weeks after radiotherapy, per subgroup

Response type	Total treated population (*n* = 432)	Patients < 68 years (*n* = 234)	All patients with breast or prostate cancer (*n* = 224)	All patients with spinal metastases^a^ (*n* = 289)	Patients in good physical condition^b^ (*n* = 202)
Responders	262 (61%)	132 (56%)	157 (70%)	172 (60%)	140 (70%)
Complete response	109 (25%)	49 (21%)	71 (32%)	72 (25%)	67 (33%)
Partial response	153 (35%)	83 (36%)	86 (38%)	100 (35%)	73 (36%)
Non-responders	128 (27%)	78 (33%)	56 (25%)	85 (29%)	53 (26%)
Stable pain	66 (16%)	42 (18%)	29 (13%)	47 (16%)	27 (13%)
Undetermined	20 (5%)	9 (4%)	7 (3%)	15 (5%)	8 (4%)
Pain progression	42 (10%)	27 (11%)	20 (9%)	23 (8%)	18 (9%)
Unknown	42 (10%)	24 (11%)	11 (5%)	32 (11%)	9 (4%)

^a^Number of patients is lower than the number of spinal lesions; some patients had more than one spinal lesion which needed radiation therapy

^b^All patients with WHO performance status of 0 or 1, indicating no or few symptoms

## Discussion

In our prospective PRESENT cohort, pain response rates were 61% in the total treated population and 67% in the assessable patients. Given the median time to response, it is important to inform patients that pain response may still occur after 4 weeks. In line with the literature, pain response for patients with spinal metastases was comparable to pain response in the entire cohort [20–22], and the best responders were patients with metastases from breast or prostate cancer and patients in good physical condition [23].

As long course radiotherapy is, in our department, reserved for patients in a (relatively) good physical condition, it was not surprising that significantly more patients experienced pain relief after long course radiotherapy. Retreatment rates in our cohort were similar between a single dose and long course radiotherapy. This is, however, not in line with randomized trials, as in the Cochrane analyses [3] retreatment rates were consequently higher after single fraction radiotherapy. Additionally, in the Dutch Bone Metastases study, time to retreatment was also shorter with a mean time to retreatment of 13 weeks after single fraction and 21 weeks after multiple fractions [23]. Irrespective of response to initial treatment, physicians are more willing to retreat after a single fraction [23]. Our retreatment rates, therefore, also reflect the selection of patients in (relatively) good physical condition when making a choice for the dose schedule.

The pattern of pain response in this unselected cohort of patients with bone metastases is comparable with response rates reported in randomized trials over the past decades. The pooled estimate of the response rates from 29 randomized controlled trials is approximately 60% [3, 4]. Of these randomized controlled trials—including at least 100 patients—just two randomized trials adhered to the international consensus guidelines for reporting outcomes [23, 24]. Pain response rates from these trials were slightly higher compared to pain response in the PRESENT cohort. Looking at the intention-to-treat population in the Dutch Bone Metastases Study, an overall response rate of 68–69% in more than 1100 patients was found [25]. Foro Arnalot et al. found response rates of 76–87% in a study population of 160 patients [24]. In this trial, patients with more than one painful site, and patients with spinal cord compression were excluded, as were patients who received prior radiotherapy or patients in an overall poor state of health. Potentially, these patients have more often progressive disease and are therefore less likely to respond to radiotherapy [2, 26]. This patient category is included in the PRESENT cohort, which may help explain the somewhat lower response rate in the current study. Another factor that could have contributed to some extent to the lower rate is the introduction of stereotactic radiotherapy in our department in November 2014. Patients eligible for stereotactic radiotherapy are recruited in the PRESENT cohort but were excluded from the current analysis. Almost 80% of the patients in our department who underwent stereotactic radiotherapy had oligometastatic disease, and more than 80% had a WHO performance status of 0 or 1. In contrast, only 53% of the patients in the cohort under study had a good performance status. As stereotactically treated patients represent a healthier subgroup, their expected response rates are higher [2, 26]. Hypothetically, adding these patients to this analysis, assuming these patients would all respond to radiotherapy, the response rate would have been near 70%.

The Dutch Bone Metastases Study is considered a landmark project since it is one of the largest studies assessing the response of patients with painful bone metastases after palliative radiotherapy [23, 25]. In this study, patients who needed treatment of more than one lesion were excluded, as were patients who were irradiated previously and had spinal cord compression, metastases in the cervical spine, or metastases from malignant melanoma or renal cell carcinoma. Patients with these characteristics were included in the PRESENT cohort and may explain the somewhat lower response rates. Furthermore, up to 3 months, pain response of the patients in the Dutch Bone Metastases Study was measured every week. Patients in the PRESENT cohort are asked to fill out questionnaires every other week, thereby increasing the chance of not recording a perceived pain response.

For PRESENT, we systematically approach all patients with bone metastases inviting them to participate. Patients are followed prospectively with extensive measures including patient-reported outcomes and clinical data for a long(er) period of time. Lost-to-follow-up rates are very low in PRESENT (10%) compared to cohorts from other hospitals (19–78%) [7, 22, 27]. Response rates after palliative radiotherapy for bone metastases very much depend on how the outcome is reported, with substantial differences between response rates based on the total treated population and those calculated in assessable patients. In addition, the follow-up period in which response is measured is of major influence with a difference in response rate of 10% between shorter and longer follow-up periods. Due to the natural course of the disease, lost-to-follow-up is very common in this study population. Patients are often admitted to the hospital or deteriorate at home, the result of which hampers the return of questionnaires leading to missing data. These missing outcomes are not likely missing at random, and simply removing patients who are lost to follow-up will most likely overestimate the proportion of patients who experienced a pain response. Accordingly, researchers should report both the response rates of their total treated population and the results of assessable patients to show the effects of this potential bias.

In 2012, the international consensus guideline was updated to include the NPR in addition to other assessments of pain response. In the PRESENT cohort, the NPR is 64% indicating that responsive patients spent around two third of their remaining life with less pain and without the need for retreatment. Probably, this is an underestimation as there are PRESENT patients who are still alive without reported pain recurrence. It is important to realize that in the last weeks before death, pain intensifies in patients with bone metastases, which has a negative impact on quality of life [28]. Although the use of the NPR is recommended for several years now, this measure is rarely reported. Only the group of Foro et al. reported the NPR, which was slightly higher compared to the NPR we have found (68–71%) [24]. In the DBMS, NPR was 70% (data not published). As the NPR includes the period of pain relief, one might argue that this outcome measure is more relevant to patients than the binary statement of response. Indeed, patients with a short period of relief do count as responders but contribute little time to the (numerator of the) NPR making this more relevant to patients.

Follow-up in our cohort included data on pain scores and analgesic use, allowing us to report our outcomes according to the international consensus guidelines [16]. However, the recommendation is to only account for opioid use when calculating response rates. In our cohort, we found that more than 30% of patients used corticosteroids during radiotherapy treatment. Corticosteroids could also have a beneficial effect on relieving pain [29]. Furthermore, patients also use tricyclic antidepressants or antiepileptic drugs for neuropathic pain. When calculating response rates, changes in the use of corticosteroids, tricyclic antidepressants, or antiepileptic drugs were not taken into account. In line with the consensus guidelines, patients with 25% decreased opioid use were counted as responders in our analyses. It is possible that patients using corticosteroids or neuropathic pain medication also had reduced intake of these analgesics as result of response after radiotherapy but were not regarded as responders.

We acknowledge that this study has some limitations. As radiotherapy is a local treatment, ideally, the response just to the index lesion is measured. However, it is difficult for patients with multiple lesions to distinguish between painful lesions at relatively small distances and give separate pain scores. As we asked patients to indicate their worst pain score, it might be that radiotherapy was successful for one lesion, but not for another. In fact, treatment to the index lesion might have *unmasked* other lesions appearing more symptomatic. Furthermore, taking analgesic use into account when calculating response is challenging because often other metastatic lesions become (more) symptomatic after the response of the irradiated metastases. In the PRESENT cohort, we enroll all patients with bone metastases, including those patients with multiple painful lesions, possibly lowering the pain response rate. However, the majority of the PRESENT patients (80%) had bone metastases treatable in one target volume. Another limitation might be our time frame of response since we calculated response up to 3 months after radiotherapy. It might be that patients who did not experience pain relief from radiotherapy started with systemic anticancer treatments, such as chemotherapy, hormonal therapy, or even radiopharmaceuticals. It might be that these systemic treatments induced a pain response which was (mistakenly) attributed to radiotherapy and pain response might thus be overestimated. However, as some patients might experience a response even after 12 weeks [30], we decided to include all responses up to 12 weeks.

## Conclusion

In our cohort, the majority of patients with painful bone metastases treated with palliative radiotherapy experienced pain relief. However, a large proportion of patients did not respond to radiotherapy. New interventions or combination of conventional treatments including radiotherapy, surgery, and systemic treatments for patients with symptomatic bone metastases should aim at improving pain relief. Before implementation in routine clinical care, these interventions are all ideally evaluated in randomized trials. The PRESENT cohort provides an infrastructure aiming at a more efficient evaluation of new (combinations of) treatments. Furthermore, it is important to identify the non-responding patients as they might benefit more from early and proactive palliative care management.
